# LEDoxy-SL: A Placebo-Controlled, Double-Blind, Randomized, 24-Month Trial of Six Weeks of Daily Doxycycline Plus Hygiene-Based Essential Care for Reducing Progression of Filarial Lymphedema in Sri Lanka

**DOI:** 10.4269/ajtmh.24-0050

**Published:** 2024-07-23

**Authors:** Thishan Channa Yahathugoda, Nirmitha Lalindi De Silva, Janaka Ruben, Sharmini Gunawardena, Mirani Vasanthamala Weerasooriya, John Horton, Philip Budge, Eric Ottesen, Sarah Mary Sullivan, Mariana Stephens, John Shen, Ute Klarmann-Schulz, Achim Hoerauf, Joseph Patrick Shott, Charles Mackenzie

**Affiliations:** ^1^ Filariasis Research Training and Service Unit, Department of Parasitology, Faculty of Medicine, University of Ruhuna, Galle, Sri Lanka;; ^2^ Department of Parasitology, Faculty of Medicine, University of Colombo, Sri Lanka;; ^3^ Tropical Projects, Hitchin, United Kingdom;; ^4^ Washington University School of Medicine, St. Louis, Missouri;; ^5^ Neglected Tropical Disease Support Center, Task Force for Global Health, Decatur. Georgia;; ^6^ Institute for Medical Microbiology, Immunology and Parasitology (IMMIP), German Centre for Infection Research (DZIF), Bonn-Cologne Site, University Hospital Bonn, Bonn, Germany;; ^7^ Division of Neglected Tropical Diseases, U.S. Agency for International Development, Washington, District of Columbia

## Abstract

Morbidity management of filarial lymphedema remains a challenge even during the post-lymphatic filariasis elimination era in Sri Lanka despite provision of the predominantly hygiene-based WHO Essential Package of Care. Because prior studies have suggested that 6 weeks of doxycycline may reduce progression of limb lymphedema, we conducted a randomized, placebo-controlled, superiority study to evaluate this possibility in Sri Lanka. Patients aged 14 to 65 years with lymphedema in one or both legs received either 200 mg of doxycycline daily for 6 weeks or matching placebo. The primary efficacy endpoint was improvement or lack of progression in lymphedema stage at 24 months postenrollment. Secondary endpoints included change in lymphedema stage at 12 and 24 months, frequency of acute adenolymphangitis episodes, and perceived disability measured by the WHO Disability Assessment Schedule 2.0 (WHODAS 2.0). Training and supplies for limb hygiene were provided throughout the study. Two hundred participants (100 in each arm) with lymphedema of Dreyer stages 1 to 3 were enrolled. By the end of the 2-year study, 29% of the doxycycline patients and 34% of those on placebo showed improvement (i.e., a decrease in lymphedema stage), whereas 11% and 15% of the two groups showed worsening of the lymphedema. Adenolymphangitis rates were comparable in the two groups (43 doxycycline and 38 placebo recipients), although attacks lasted slightly longer in placebo patients (6.5 days versus 5.2 days). In both groups, perceived disability improved initially, with partial rebound in the second year. Only 34 adverse events affecting 24 patients (11%) occurred during the 6-week treatment period. Although doxycycline did not significantly impact lymphedema progression in this study, the results clearly indicate that clinical and personal benefits can be obtained from intensive hygiene management alone.

## INTRODUCTION

Adult filarial parasites of *Wuchereria bancrofti* and *Brugia malayi* cause lymphatic filariasis (LF) in humans. These filarial nematodes, especially dead or dying worms, cause inflammation and dysfunction of the lymphatic system, leading to chronic debilitating conditions such as lymphedema, hydrocele, lymph scrotum, and chyluria.^1^^,^^2^ Secondary bacterial infections cause episodes of acute adenolymphangitis (ADL) that progressively worsen the disease condition and can finally result in elephantiasis. In July 2016,^3^ the WHO certified Sri Lanka as having eliminated LF as a public health problem through repeated mass drug administration (MDA) programs using albendazole and diethylcarbamazine (DEC). However, morbidity management and disability prevention (MMDP) needs from the earlier infections remain persistent challenges for the national antifilariasis campaign during the postelimination period in the country.

Current treatment practices for lymphedema rely on decreasing the number of acute attacks by improving the hygiene of affected limbs, use of appropriate topical antibiotics and antifungals, exercise, elevation of the affected limb, and the use of proper footwear, recognized as the Essential Package of Care (EPC) by the WHO.^4^^,^^5^ Such hygiene-based lymphedema management, either alone or in combination with prophylactic oral penicillin, has clearly demonstrated the ability to control, or even reverse, disease progression,^5^^–^^7^ but it does require sustained access to resources required for limb care and strict adherence to the prescribed procedures. Hygiene-based MMDP is best achieved through daily reinforcement by health personnel (“daily touch and talk” strategy), with advice on maintenance of limb hygiene and treatment of entry lesions.^6^ However, such individual daily attention from a designated health officer is generally not feasible, and only about one-third of affected patients actively adhere to this approach.^4^^,^^5^

Doxycycline’s macrofilaricidal effect on filarial parasites that harbor the bacterial endosymbiont *Wolbachia* have been previously documented.^8^^,^^9^ A 6-week course of doxycycline (200 mg daily) has also been reported to be associated with reduced disease progression in lymphedema patients with or without active infection with *W. bancrofti*,^10^^–^^12^ an observation potentially related to doxycycline’s potential role in inhibiting lymphatic endothelium-derived vascular endothelial growth factor-C and other angiopoietin-like factors involved in the pathogenesis of lymphedema.^10^^,^^11^

To test the hypothesis that a 6-week course of doxycycline reduces progression of limb lymphedema, regardless of active filarial infection, a consortium led by the Task Force for Global Health (TFGH) in Atlanta, Georgia, and the University of Bonn (Germany) initiated a series of coordinated but independently powered clinical trials to evaluate the efficacy of this approach in diverse geographic and community settings.^13^ We report here the results of the first initiated study site in Sri Lanka. The trial was, importantly, successfully conducted during the COVID-19 pandemic, a trying period in which numerous challenges were thrust upon populations worldwide, including some that substantially affected the procedures for conducting clinical trials in safe and effective ways.^14^^,^^15^ The challenges faced during this period of the trial are also highlighted in this report.

## MATERIALS AND METHODS

### Trial design.

LEDoxy-Sri Lanka was a randomized and controlled observer-, provider-, and patient-blinded superiority trial with two parallel study groups and a primary endpoint of change in the stage of patients’ lymphedema at 24 months. The overall design of the study has been published in detail elsewhere.^13^

### Ethics statement.

The protocol was approved by the Western Institutional Review Board (Olympia, WA) and additionally by the Ethics Committee, Faculty of Medicine, University of Colombo (Colombo, Sri Lanka). The study was registered on ClinicalTrials.gov (Sri Lanka: clinical trial number NCT02929134). Informed written consent was obtained from all individuals.

### Study setting.

In accordance with the guidelines of WHO’s Global Program to Eliminate Lymphatic Filariasis (GPELF), the Government of Sri Lanka initiated its national program (National PELF) under the purview of the Ministry of Health in 2002. Annual MDA with DEC and albendazole was given to the entire endemic population in the country between 2002 and 2006. The Filariasis Research Training and Service Unit (FRTSU) of the University of Ruhuna formed the main research arm of the National PELF. It was an independent group to monitor and evaluate the posttreatment surveillance activities. After the successful interruption of transmission in 2016, the WHO formally acknowledged the elimination of LF as a public health problem in Sri Lanka.^3^^,^^16^

Participants for the current study were recruited from coastal villages in the Galle and Matara districts of southern Sri Lanka, an area well characterized by the clinical burden of filariasis.^17^^,^^18^ National guidelines for lymphedema management reflect the WHO’s EPC recommendations for the use of appropriate hygiene care (regular washing with soap and water and skin and nail care), use of topical antibiotics and antifungals as needed, regular exercise, elevation of the affected limb, the use of proper footwear, and for patients with lymphedema grade 3 and above,^4^ daily oral penicillin given routinely for lifelong prophylaxis.^16^

### Recruitment.

**Figure 1.**
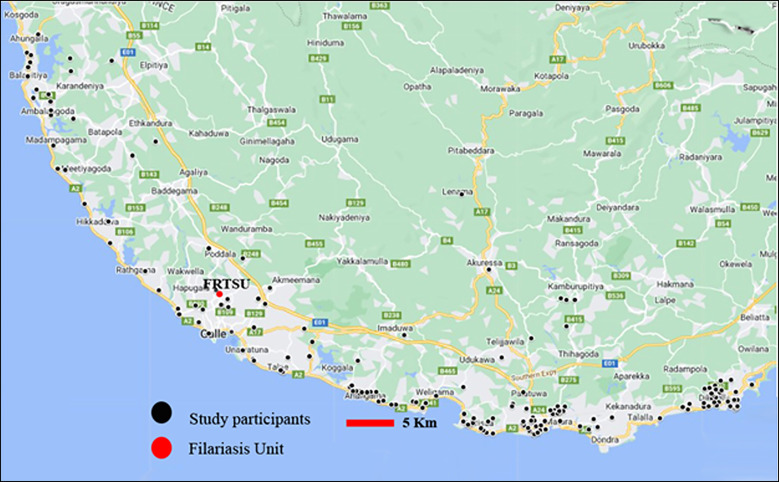
Location of study area in Sri Lanka and of participants in relation to the Filariasis Unit in Galle. FRTSU = Filariasis Research Training and Service Unit.

Participants were recruited from the Galle and Matara districts and their adjacent suburbs: Unawatuna, Gintota, Bope, Balapitiya, Ambalangoda, Hikkaduwa, Walgama, Pamburana, Madihe, Polhena, Dikwella (Figure 1). We used a snowball recruitment method; participants receiving care at the FRTSU clinic were invited to participate, and loudspeaker announcements were made in the selected areas. Participants and households contacted by these methods were also asked to refer other lymphedema patients in their communities.

### Eligibility, consent, and randomization.

Inclusion and exclusion criteria have been published in detail previously.^13^ Briefly, eligible patients were men and nonpregnant, nonbreastfeeding women aged 14 to 65 years with lower extremity lymphedema (Dreyer stage 1 to 3)^4^ in one or both legs. Women of childbearing potential were required to adhere to an approved and effective method of contraception before, during, and for at least 2 weeks after the doxycycline or placebo treatment period. All participants provided written informed consent prior to any study procedures. Participants were randomized in a 1:1 ratio to receive either 200 mg of doxycycline or a matching placebo daily for 6 weeks. Doxycycline procurement, placebo production, packaging, randomization, and shipping details have been described elsewhere.^13^ Randomization was stratified according to the lymphedema stage of the more affected leg of the participants (early grades 1 to 3). For reasons of comparison, persons with more advanced lymphedema (late grades 4 to 6) were not excluded from participating in the study program. However, their enrollment stopped when the target number of 100 lymphedema patients of grades 1 to 3 in each arm of the study was reached; analysis of their data (referred to as group B) will be performed and reported separately because the number of such patients was relatively low (∼10 patients per study arm).

### Screening, enrollment, assessment, and follow-up.

After the acquisition of informed consent for screening, data collected at screening included a detailed history of lymphedema, including duration of disease, number and description of acute ADL episodes, history of treatment with antifilarial drugs during MDA, and details of other concomitant treatments. Participants were examined for lymphedema, hydrocele, and other morbidities related to LF. Laboratory evaluation included complete blood count, liver function tests (aspartate aminotransferase/alanine transaminase/gamma-glutamyl transferase, renal function tests (serum creatinine, blood urea nitrogen), urine pregnancy test (for women of childbearing potential), and circulating filarial antigen detection by Filariasis Test Strip (Alere, Scarborough, ME). Circulating filarial antigen-positive individuals were reexamined with a filtration technique using a night blood sample. The pregnancy test was repeated on treatment day 1 (enrollment day) and again 2 weeks after completion of treatment.

### Lymphedema staging.

Staging of the lymphedema was done at each contact point using the seven-stage classification described by Dreyer et al.^4^ To further document the stage, digital photographs of both limbs were taken at baseline and at each follow-up visit (6, 12, 18, and 24 months). Images were taken from four directions, and the distance, lighting, and background were standardized. Close-ups were taken to show mossy lesions, nodules, and deep skin creases.

### Limb measurements.

A tape measure was used to determine leg circumference. A designated medical officer (MO) measured the circumferences of each leg with a tape while the patient was in a seated position. Circumference measurements were taken at 10 cm posterior to the tip of the large toe and at 12, 20, and 30 cm from the sole. Each measurement was taken twice, and the average was used in the analysis. Measurement of skin thickness was done by ultrasound according to a standard protocol^19^ using a portable hand-carried ultrasound system equipped with a 38-mm 5- to 10-MHz linear-array transducer (Lumify probe; Philips, Seattle, WA). Limb volume was measured as described previously, using the portable LymphaTech three-dimensional infrared imaging system (LymphaTech, Atlanta, GA).^19^ All limb measurements were taken between 2 pm and 7 pm. All scans were performed during this same time period at every visit, to ensure consistency throughout the trial.

### Quality of life and hygiene status assessments.

Quality of life assessments were performed at baseline and at 12 and 24 months, using a translated version of the WHO Disability Assessment Schedule 2.0 (WHODAS 2.0),^20^ a generic health and disability assessment tool grounded in the conceptual framework of the International Classification of Functioning, Disability, and Health and capturing an individual’s level of function in six major life domains, half of these related to physical disabilities potentially associated with LF and half to nonphysical potential disabilities. A specially trained MO was designated to administer the Sinhala version of WHODAS 2.0 to participants. The Sinhala version of WHODAS 2.0 was developed and linguistically validated prior to administration. Two additional questionnaires were used to assess the hygiene status of participants: one was used to obtain information regarding limb hygiene and to explore the frequency of washing, cleaning, drying, and care provided for nails, entry lesions, and the skin, and the other was used to examine the limbs to observe the hygiene to identify general cleanliness, nail management, interdigital skin status, sole cracks, and presence of open wounds. All questionnaires had standardized scoring systems to quantify the qualitative data.

### Minimum care package (MCP) for hygiene.

All patients were trained to clean the affected limb based on the principles outlined in the “New Hope” booklet for persons with lymphedema.^7^ A simplified one-page handout was prepared with attractive cartoons in the local language (Sinhala) and shared with patients. In addition, a qualified physician visited each patient at 3, 6, 12, and 18 months to monitor and improve the MCP applications at each contact point. The standardized MCP included 1) cleaning the affected limb daily with soap and water, 2) keeping the affected limb dry, 3) clipping of the nails, 4) using appropriate antibiotics for ADL episodes, 5) applying antifungal ointment to webs of the toes, nails, and sides of the feet every night, 6) elevation of the affected extremity, 7) limb exercises as instructed, and 8) encouraging and monitoring the use of appropriate footwear. Each patient received soap, towels, plastic bowls for washing the limbs, a plastic stool to keep the limb elevated, informational material in Sinhala, and a diary to record ADL attacks. The participants were provided adequate amounts of soap and towels at each contact point.

### Study drug administration and monitoring.

Doxycycline or matching placebo was administered under supervision (directly observed treatment [DOT]) for 6 weeks. Medication was administered with meals together with 250 mL of water, and the participants were advised to sit up for at least 30 minutes after ingestion to reduce the risk of esophageal irritation and ulceration. Any vomited doses were replaced. A field-based DOT strategy was adopted. Preintern, qualified MOs were selected as the treatment providers; in some cases, MOs were assisted by Public Health Field Officers (PHFO). The MO or PHFO visited the patient’s home daily to administer the medication, assess adverse events, and record any concomitant medications. The patient or a family member maintained a diary, and each MO or PHFO checked all diaries weekly. The MO managed all ADL attacks during the 6-week treatment period using a standardized protocol, including antipyretic, anti-inflammatory, oral antibiotic, topical antibiotic, or antifungal medication, as indicated. Queries or adverse events requiring further attention were referred to a single responsible physician. If any participant was unable to consume the drugs under observation because of unavailability at home or traveling away from home, they were given the necessary quota of drugs and instructed to take them while on a video call with either the designated MO or PHFO.

### Concomitant care and interventions during the trial.

Clinical information about patients who were treated by MOs other than the study doctors for any concurrent illnesses was collected by various methods. Using the patient’s identification card, the family doctor contacted the principal investigator or supervising MO just before managing the patient, thereby preventing any possible drug duplications and/or interactions. All medications and clinical events were recorded in the patient’s diary. During the DOT and throughout the 24 months of follow-up, participants were not given antifilarial drugs except for two filarial antigen-positive individuals identified at baseline. However, those who were on a previously prescribed oral penicillin prophylaxis regimen either abandoned or continued according to their will. Short courses of antibiotics (mainly amoxicillin/clavulanate, amoxicillin, cloxacillin, and flucloxacillin) were permitted to treat ADL attacks and other concurrent conditions such as wounds and urinary or upper respiratory tract infections.

### Data collection, management, and analysis plan.

Data were captured using clinical report forms especially designed and approved for the study. Research data were captured using mixed methods, paper and electronic. Research data collected on paper clinical report forms were then entered into the REDCap^®^ electronic data capture tools hosted at Emory University.^21^^,^^22^ REDCap (Research Electronic Data Capture) is a secure, web-based software platform designed to support data capture for research studies by providing 1) an intuitive interface for validated data capture, 2) audit trails for tracking data manipulation and export procedures, 3) automated export procedures for seamless data downloads to common statistical packages, and 4) procedures for data integration and interoperability with external sources. All electronic tools used for data collection were password protected.

Sample size calculations and the statistical analysis plan have been previously described in detail.^13^ Primary efficacy endpoints were the improvement or lack of progression of lymphedema stage when examined 24 months after onset of treatment. Prespecified secondary endpoints included the following: 1) change of lymphedema stage (reduction or increase) compared with baseline assessed at 6, 12, 18 and 24 months; 2) change in the circumference of the affected limb from baseline; 3) reduction in the frequency of ADL attacks evaluated from 0 to 12 months and from 12 to 24 months after onset of treatment; and 4) changes in skin thickness at 12 and 24 months compared with the baseline.

Descriptive statistics were calculated for all baseline variables, and bivariate analyses were conducted to determine if randomization yielded treatment and control groups with similar characteristics at baseline. Frequencies were calculated for categorical variables, and Fisher’s exact tests were used. For continuous variables, descriptive statistics were calculated (mean, standard deviation, minimum, maximum, range), and *t* tests were performed to assess differences between the doxycycline and placebo groups. For continuous variables that were non-normal, nonparametric statistics were used.

Outcomes at 6, 12, and 24 months were assessed for differences between doxycycline and placebo groups. Differences between doxycycline and placebo groups were compared using two-sided hypotheses with α = 0.05 and 95% CI. Categorical variables were analyzed using Fisher’s exact tests. Ordinal variables, such as whether the lymphedema stage progressed, stayed the same, or improved, were assessed using the Jonckheere-Terpstra test for trend. Kaplan-Meier curves were used to visualize the time to the first ADL attack, and median “survival times” were calculated.

Mixed-effects models with a time effect were used to determine whether differences in the measurement endpoints exist between study groups over time. Study group and time were the main effects. Baseline characteristics, e.g., sex, age, and disease history, were included as covariates. Survival analysis techniques were used to assess whether differences in time to an acute attack exist between the placebo and treatment groups. Cox proportional hazard regression analysis was used to assess the impact of the treatment adjusted for other covariates.

All analyses were conducted on the intention-to-treat population. Statistical analyses were performed using SAS 9.4 (SAS, Cary, NC), R Statistical Software (v. 4.3.1),^23^ and the ggplot2 package.^24^

## RESULTS

### Characteristics of the study population and its lymphedema presentation.

**Figure 2.**
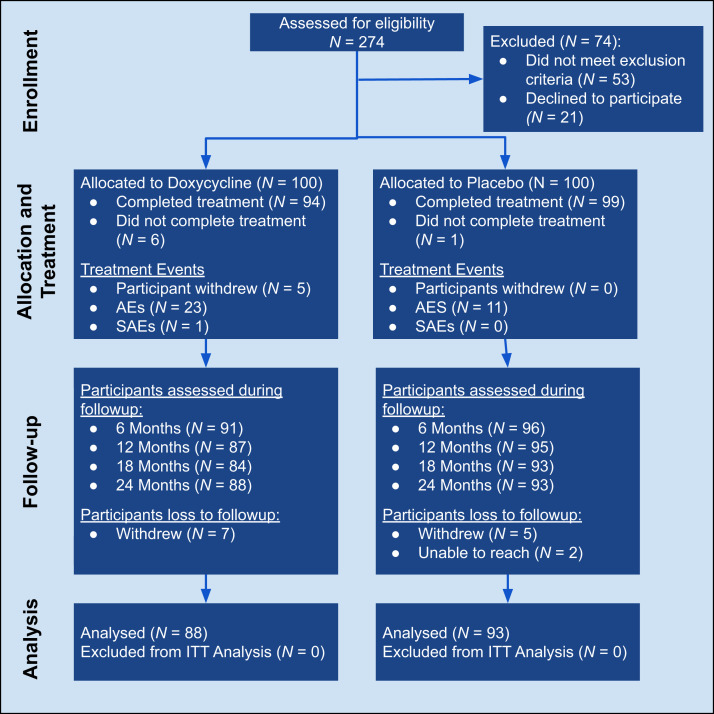
Study flow. AEs = adverse events; ITT = intention to treat; SAEs = serious adverse events.

Two hundred seventy-four patients were screened for eligibility to achieve the enrollment goal of 100 participants with lymphedema stages 1 to 3 in each study arm (Figure 2; Table 1). The majority were female (70%; *N* = 140) with, the mean body weight was 69 kg, and all participants had lived in areas where LF was endemic for at least 5 years, with a median duration of 35 years (range, 5–65 years). Only 19% (*N* = 38) recalled having taken the antifilarial medication provided through the national MDA program, whereas 53% (*N* = 106) said they did not. Only two individuals were serologically positive for filarial antigen, and neither of them was microfilaria positive on blood examination.

**Table 1:** Baseline: participant characteristics by study arm

Characteristics	DOX 200	Placebo	*P*-Value (test)
Total participants (*N*)	100	100	
Sex, *n* (%)			
Male	29 (29%)	31 (31%)	0.8775 (Fisher)
Female	71 (71%)	69 (69%)	
Age (years), mean ± SEM (95% CI), min–max	50.85 ± 0.90 (49.07–52.63), 22–64	48.04 ± 1.07 (45.92–50.16), 14–64	0.0454 (*t* test)
Body weight (kg), mean ± SEM (95% CI), min–max	69.90 ± 1.45 (67.02–72.78), 40–130	68.03 ± 1.26 (65.53–70.53), 43–103	0.3326 (*t* test)
Body weight, *n* (%)			
≤50 kg	7 (7%)	6 (6%)	1.0000 (Fisher)
>50 kg	93 (93%)	94 (94%)	
No. of years in area of endemicity, mean ± SEM (95% CI), min–max	36.93 ± 1.67 (33.62–40.24), 5–65	35.65 ± 1.61 (32.46–38.84), 9–64	0.5810 (*t* test
Prior MDA, *n* (%)			
Yes	20 (26.32%)	18 (26.47%)	1.0000 (Fisher)
No	56 (73.68%)	50 (73.53%)	
Unknown	24 (24%)	32 (32%)	
FTS, *n* (%)			
Positive	2 (2.06%)	0 (100%)	0.4975 (Fisher)
Negative	95 (97.94%)	94 (100%)	
Microfilariae (in FTS positives), *n* (%)			
Positive	–	–	NA (Fisher)
Negative	2 (100%)	–	

DOX 200 = group receiving 200 mg of doxycycline daily for 6 weeks; FTS = filaria test strip; MDA = mass drug administration; min–max = minimum–maximum; placebo = group receiving placebo daily for 6 weeks; SEM = standard error of the mean.

Lymphedema presentation among the study patients did not differ significantly between the doxycycline and placebo groups (Table 2), with the distribution of lymphedema stages between the two groups being similar, as well as the proportion of patients with a single leg or with both legs affected. By history, the numbers of years that the patients had suffered from ADL attacks before the study, the numbers of months since the last prior attack, and both the frequencies and durations of the attacks during the past year were similar for the two study groups. Also, at baseline, the cleanliness and observer-assessed skin scores were comparable for the two groups of patients.

**Table 2:** Baseline: LE presentation and management

Characteristics	Doxycycline	Placebo	*P*-Value (test)
LE present for indicated years, *N*	100	99	**0.8715** (*t* test)
Mean ± SEM (95% CI)	11.01 ± 1.09 (8.85, 13.17)	10.74 ± 1.29 (8.18, 13.29)	
Median, min–max	6.00, 0.08–41.00	6.00, 0.08–63.00	
LE stage, right leg			
Total *N* (%)	100 (100%)	100 (100%)	
Stage 0	28 (28%)	26 (26%)	
Stage 1	14 (14%)	19 (19%)	
Stage 2	27 (27%)	34 (34%)	
Stage 3	28 (28%)	20 (20%)	
Stage 4	1 (1%)	0 (0%)	
Stage 5	1 (1%)	0 (0%)	
Stage 6	1 (1%)	1 (1%)	
Stage 7	0 (0%)	0 (0%)	
LE stage, left leg			
Total *N* (%)	99 (100%)	100 (100%)	
Stage 0	25 (25%)	30 (30%)	
Stage 1	15 (15%)	15 (15%)	
Stage 2	27 (27%)	20 (20%)	
Stage 3	30 (30%)	32 (32%)	
Stage 4	0 (0%)	0 (0%)	
Stage 5	0 (0%)	2 (2%)	
Stage 6	2 (2%)	1 (1%)	
Stage 7	0 (0%)	0 (0%)	
Legs affected, *n* (%)			
Both legs	46 (46%)	44 (44%)	**0.8870** (Fisher)
Only one leg	54 (54%)	56 (56%)	
ADL attacks for number of years prestudy, *n* (%)			
<1 year	8 (8%)	9 (9%)	
1 to <5 years	27 (27%)	18 (18%)	
5 to <10 years	8 (8%)	13 (13%)	
10 to <15 years	6 (6%)	7 (7%)	
15 to <20 years	3 (1%)	1 (1%)	
≥20 years	9 (9%)	12 (12%)	
Unknown	39 (39%)	40 (40%)	
Last ADL attack (months prestudy)			
N*	71	69	
Mean ± SEM, min–max	38.96 ± 7.79, 0–376	40.61 ± 8.82, 0–446	**0.8884** (*t*-test)
Duration of last ADL attack (days)			
N*	72	70	
Mean ± SEM (95% CI), min–max	6.88 ± 0.58 (5.73–8.02), 1–30	7.39 ± 0.98 (5.43–9.35), 2–60	**0.6546** (*t*-test) (unequal variance)
Number of attacks within the last year			
N*	66	65	
Mean ± SEM (95% CI), min–max	0.76 ± 0.15 (0.46–1.06), 0–6	1.00 ± 0.21 (0.58–1.42), 0–10	**0.3530** (*t*-test) (unequal variance)
Duration of attacks (avg days in past year)			
N*	37	40	
Mean ± SEM (95% CI), min–max	5.30 ± 1.04 (3.18–7.41), 0–30	5.25 ± 0.90 (3.44–7.06), 0–30	**0.9725** (*t*-test)
Observer-assessed cleanliness score			
N*	100	100	
Mean ± SEM (95% CI), min–max	0.97 ± 0.01 (0.95–0.99), 0.33–1	0.97 ± 0.01 (0.95–0.98), 0.67–1	**1.0000** (*t*-test)
Observer-assessed skin score			
N*	100	100	
Mean ± SEM (95% CI), min–max	0.93 ± 0.01 (0.92–0.95), 0.67–1	0.94 ± 0.01 (0.92–0.95), 0.67–1	**0.5435** (*t*-test)
Overall hygiene: limb kept washed and clean, *n* (%)	72 (73%)	80 (81%)	**0.2386** (Fisher)

ADL = acute adenolymphangitis; LE = lymphedema; min–max = minimum–maximum; N* = Total participants; SEM = standard error of the mean.

### Effects of treatment on lymphedema.

**Figure 3.**
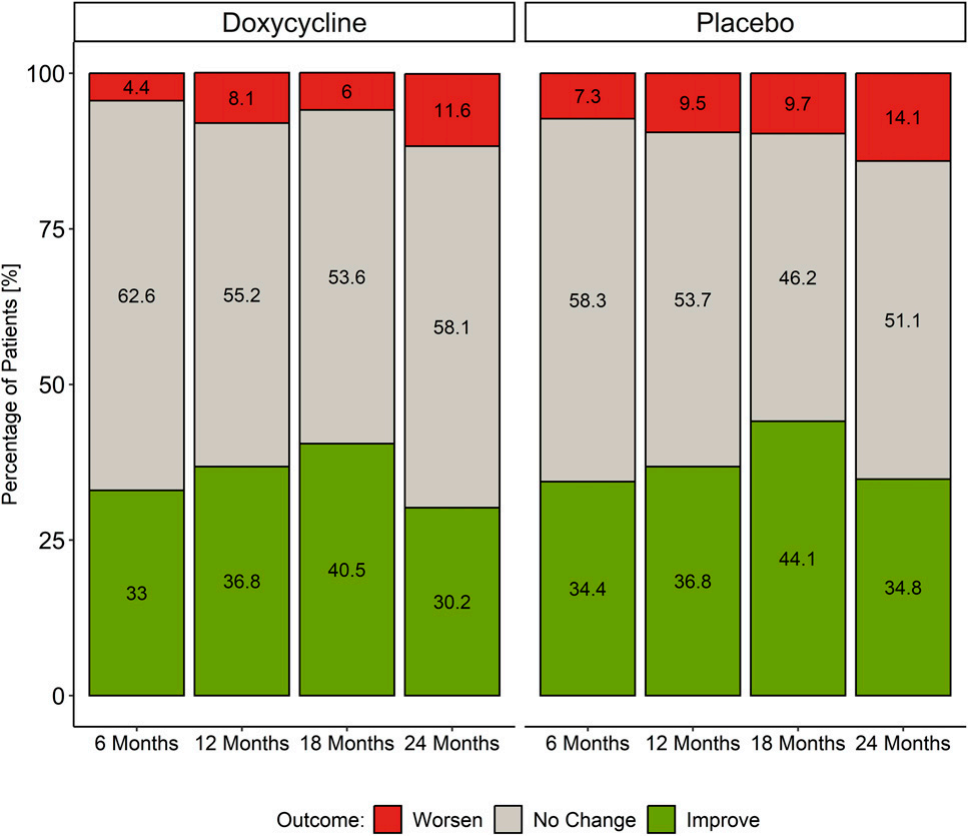
Proportion (%) of participants showing improvement, no change, and worsening in lymphedema staging at 6, 12, 18, and 24 months follow-up.

**Figure 4.**
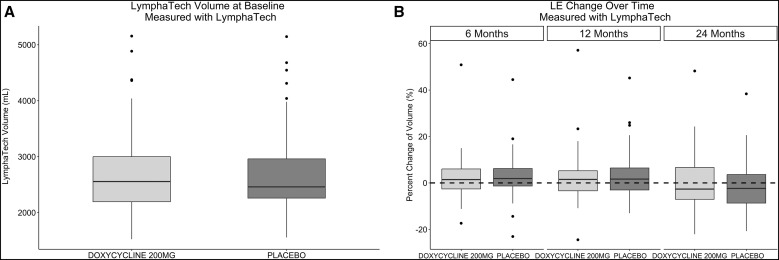
Limb volume changes over time. Baseline limb volumes (**A**) were not significantly different between doxycycline (light shading) and placebo (dark shading) groups, and limb volume changes (**B**) did not differ between doxycycline (light) and placebo (dark bars) treatment groups over time. LE = lymphedema.

**Figure 5.**
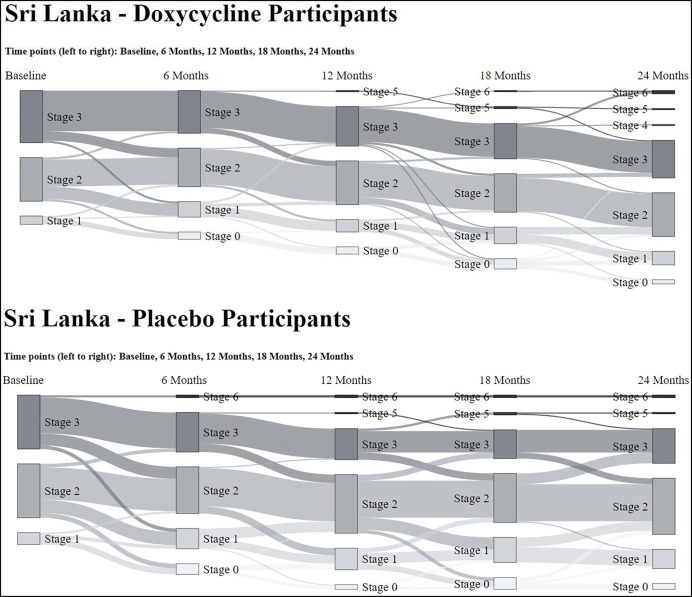
Sankey diagram showing progression across stages in doxycycline and placebo groups.

The effects of the two treatment regimens were determined as changes in each individual’s lymphedema stage at study months 6, 12, 18, and 24. The patterns of change over time (worsening, no change, or improvement) were very similar for both the doxycycline and placebo treatment groups (Figure 3; Table 3). By the end of the 2-year study, 29% of the doxycycline patients and 34% of those on placebo showed improvement (i.e., a decrease in lymphedema stage), while 11% and 15% of the two groups showed worsening of the lymphedema. The primary outcome target of this study had been to discriminate between the progression (worsening) of patients’ lymphedema stage in comparison with “improvement” or “no worsening.” No significant difference was seen, however, between the effects of the two regimens (Table 4). To assess whether any impact of doxycycline treatment could have been masked by the routine administration of penicillin to participants with stage 3 lymphedema, the primary analysis was repeated in a stratified manner, but no difference was seen between those stage 3 patients who received daily prophylactic penicillin and those who did not. Similarly, examination of other secondary outcomes of the study (lymphedema improvement versus lack of improvement, changes in limb volume [Figure 4] or limb circumference [data not shown]) again showed no significant difference between the doxycycline and placebo groups. Interestingly, there was considerable variability within the groups, with some participants at each time point in each study arm experiencing lymphedema progression (worsening) and others experiencing improvement. This interindividual variability can be appreciated more easily when the data are presented as a Sankey diagram, which shows the proportion of patients changing lymphedema grade over time (Figure 5).

**Table 3:** LE stage changes (compared with baseline)

Group	Outcome	6 Months	12 Months	18 Months	24 Months
DOX 200	Outcomes, *N*	91	87	84	89
	Improvement, *n* (%)	29 (31.9%)	31 (35.6%)	34 (40.5%)	26 (29.2%)
	No change, *n* (%)	58 (63.7%)	49 (56.3%)	45 (53.6%)	53 (59.6%)
	Worsening, *n* (%)	4 (4.4%)	7 (8.1%)	5 (6.0%)	10 (11.2%)
Placebo	Outcomes, *N*	96	95	93	93
	Improvement, *n* (%)	31 (33.0%)	35 (36.8%)	41 (44.1%)	32 (34.4%)
	No change, *n* (%)	58 (60.4%)	51 (53.7%)	43 (46.2%)	47 (50.5%)
	Worsening, *n* (%)	7 (7.3%)	9 (9.5%)	9 (9.68%)	14 (15.1%)
	Comparison to placebo, *P*-value (Jonckheere-Terpstra test)	0.8385	0.9980	0.8751	0.8183

DOX 200 = group receiving 200 mg of doxycycline daily for 6 weeks; LE = lymphedema; placebo = group receiving placebo daily for 6 weeks.

**Table 4:** Primary outcome: LE progression (worsening) vs. absence of progression (i.e., improvement or no change)

Group	Status	6 Months	12 Months	18 Months	24 Months
DOX 200	Progression (worsening) of LE, *n* (%)	4 (4.4%)	7 (8.1%)	5 (6.0%)	10 (11.2%)
	Absence of progression, *n* (%)	87 (95.6%)	80 (92.0%)	79 (94.1%)	79 (88.8%)
Placebo	Progression (worsening) of LE, *n* (%)	7 (7.3%)	9 (9.5%)	9 (9.7%)	14 (15.1%)
	Absence of progression, *n* (%)	89 (92.7%)	86 (90.5%)	84 (90.3%)	79 (85.0%)
	Comparison to placebo, *P*-value (Fisher)	0.5380	0.7978	0.4137	0.5146

DOX 200 = group receiving 200 mg of doxycycline daily for 6 weeks; LE = lymphedema; placebo = group receiving placebo daily for 6 weeks.

### ADL episodes, hygiene, and perceived disability.

**Figure 6.**
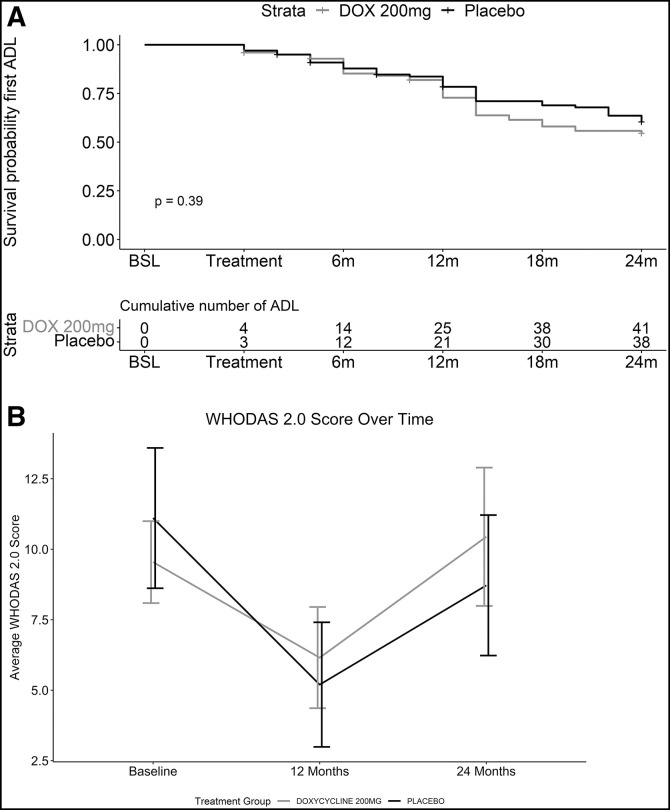
Acute adenolymphangitis (ADL) episodes and perceived disability. (**A**) Cumulative number of ADL episodes did not differ over time between doxycycline (DOX; dark line) and placebo (light line) participants. BSL = baseline. (**B**) Perceived disability, as measured by the WHO Disability Assessment Schedule (WHODAS) 2.0 survey, was reduced at 12 months posttreatment in both groups but did not differ between doxycycline (light line) and placebo (dark line) participants.

Acute adenolymphangitis episodes are a significant driver of lymphedema progression, and their prevention is a principal goal of hygiene-based lymphedema management. Prevention of ADL episodes might be a possible mechanism by which doxycycline, a broad-spectrum antibiotic with good distribution to the skin, could prevent lymphedema progression. However, in measuring the time between doxycycline or placebo treatment and either the first ADL episodes or the overall rates of ADL episodes over the course of the study, no differences were seen between the two treatments (Figure 6A). The average duration of an ADL attack was 5.2 days at 24 months in the doxycycline arm, while it was longer (6.5 days) for the placebo arm, although the difference was not statistically significant.

To document the effect of study interventions on perceived disability, we administered the WHODAS 2.0 to participants at baseline (Table 5) and at 12 and 24 months (Figure 6B). Participants in both groups experienced reductions in perceived disability during the first year of the study, but with rebounds towards baseline at 24 months and no significant differences in WHODAS 2.0 scores between groups at any time point.

**Table 5:** WHODAS 2.0 scores

Treatment Group		Baseline	12 Months	24 Months
DOX 200	*N*	100	87	86
	Mean ± SEM (95% CI)	9.54 ± 0.73 (8.09–10.99)	6.15 ± 0.90 (4.36–7.95)	10.44 ± 1.23 (7.99–12.89)
	Median, min–max	8.33, 0–35.42	4.17, 0–43.75	8.33, 0–60.42
Placebo	*N*	100	95	92
	Mean ± SEM (95% CI)	11.10 ± 1.25 (8.62–13.59)	5.20 ± 1.11 (2.99–7.41)	8.72 ± 1.25 (6.23–11.21)
	Median, min–max	8.33, 0–81.25	2.08, 0–70.83	4.17, 0–85.42

DOX 200 = group receiving 200 mg of doxycycline daily for 6 weeks; min–max = minimum–maximum; placebo = group receiving placebo daily for 6 weeks; SEM = standard error of the mean.

#### Hygiene.

Hygiene is believed to be particularly important in determining ADL episodes and lymphedema progression, so documenting adherence to hygiene was key to understanding participants’ experiences and interpreting their outcomes. Hygiene scores in the placebo arm were better than those in the doxycycline arm throughout the study (Table 6). Hygiene scores in the doxycycline arm were lower than those in the placebo group at baseline and throughout most follow-up time points. The difference between them was only slight during midstudy but increased significantly at the 24-month time point for reasons that remain unclear but that are possibly related to patients’ perception of improvement and, therefore, less concern about maintaining scrupulous attention to elements of the limb hygiene treatment program.

**Table 6:** Observer-assessed hygiene over time*

Treatment Group	Hygiene	4 Months	6 Months	12 Months	18 Months	24 Months
DOX 200	Limb kept washed and clean, *n* (%)	87 (95.6%)	89 (97.8%)	84 (98.8%)	79 (94.1%)	64 (71.9%)
	Limb not kept washed and clean, *n* (%)	4 (4.4%)	2 (2.2%)	1 (1.2%)	5 (5.95%)	25 (28.1%)
Placebo	Limb kept washed and clean, *n* (%)	93 (95.9%)	92 (98.9%)	93 (98.9%)	91 (97.9%)	85 (91.4%)
	Limb not kept washed and clean, *n* (%)	4 (4.1%)	1 (1.1%)	1 (1.1%)	2 (2.2%)	8 (8.6%)
	Comparison to placebo, *P*-value (Fisher)	1.0000	0.6189	1.0000	0.2589	0.0009

DOX 200 = group receiving 200 mg of doxycycline daily for 6 weeks; placebo = group receiving placebo daily for 6 weeks.

* Over the course of the study, including baseline, a higher proportion of participants in the placebo group were assessed to have washed and clean limbs. However, this difference was statistically significant only at the 24-month visit.

### Predictors of progression (worsening).

**Figure 7.**
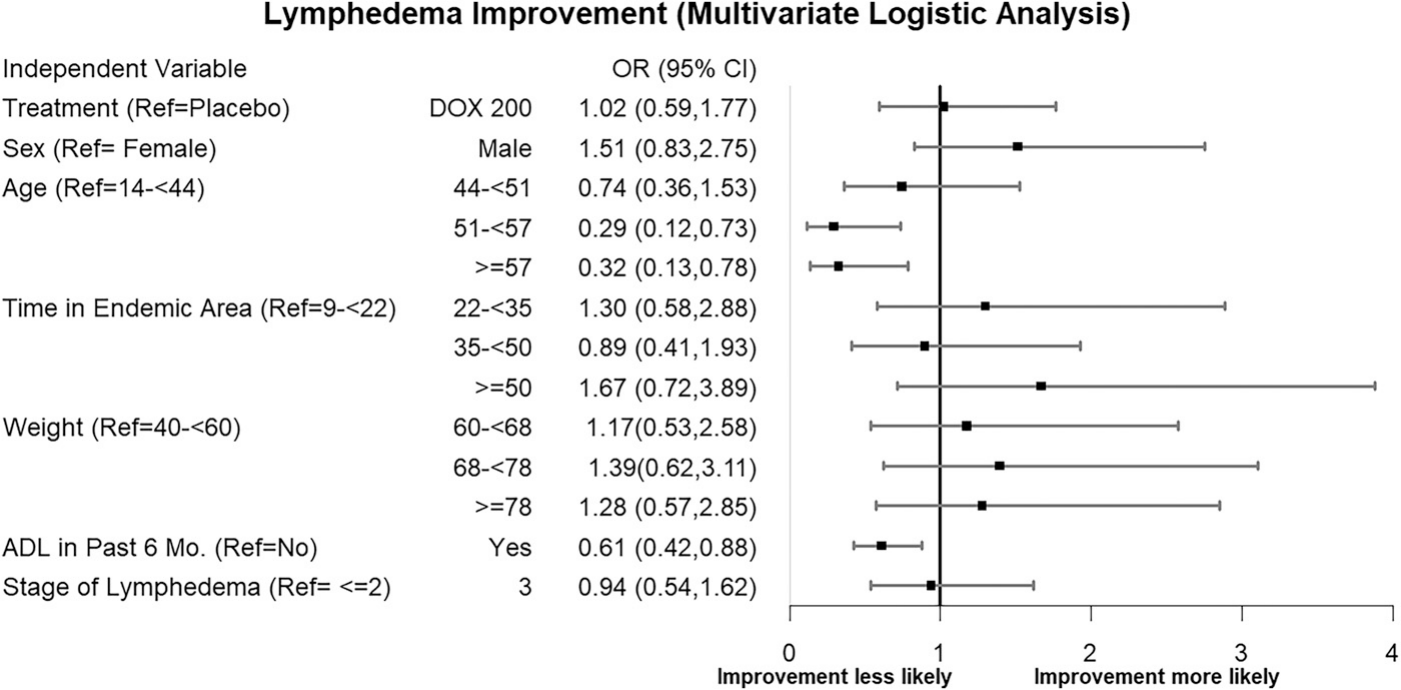
Predictors of improvement in lymphedema stage. This forest plot graphs the odds ratios (OR) and corresponding CI of the fixed effects in a mixed-effects model whose outcome was reduced lymphedema stage across the study (measured repeatedly at 6, 12, 18, and 24 months). While the CI of most predictors crossed an OR of 1, indicating no significant effect, age greater than 51 years (compared with the referent of age less than 44 years) and having experienced acute adenolymphangitis (ADL) in the past 6 months were associated with a lower likelihood of reduction in lymphedema stage. DOX = doxycycline; Ref = reference.

**Figure 8.**
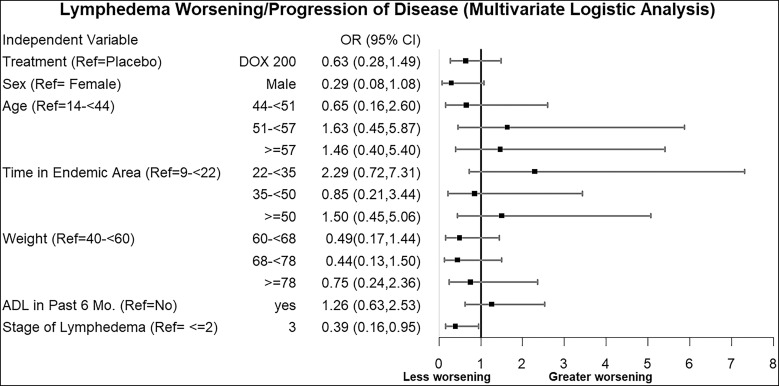
Predictors of lymphedema worsening (progression of disease). This forest plot graphs the odds ratios (OR) and corresponding CI of the fixed effects in a mixed-effects model whose outcome was increased/progressed lymphedema stage across the study (measured repeatedly at 6, 12, 18, and 24 months). While the CI of most predictors crossed an OR of 1, indicating no significant effect, the baseline lymphedema stage was the only significant predictor of stage progression, with baseline stage 3 limbs less likely to progress than limbs at stages 1 and 2. ADL = acute adenolymphangitis; DOX = doxycycline; Ref = reference.

Although there were no significant differences between the doxycycline and placebo groups in the prespecified outcomes described above, there was marked variability among individuals in each group. We therefore conducted multivariate regression analyses to identify risk factors for worsening of lymphedema among study participants across the follow-up time points. Significant predictors of worsening outcome during the study were the following: 1) being aged 51 years and above, compared with those aged between 14 and 44 years, and 2) having experienced ADL during the 6 months prior to the study (Figure 7). When the relation between baseline lymphedema stage and stage progression was examined, only stage 3 limbs showed a significant relationship, and that was being less likely to worsen than limbs of stages 1 and 2 (odds ratio 0.39; 95% CI, 0.16–0.95). There was also a trend towards less worsening among men than among women (Figure 8).

**Figure 9.**
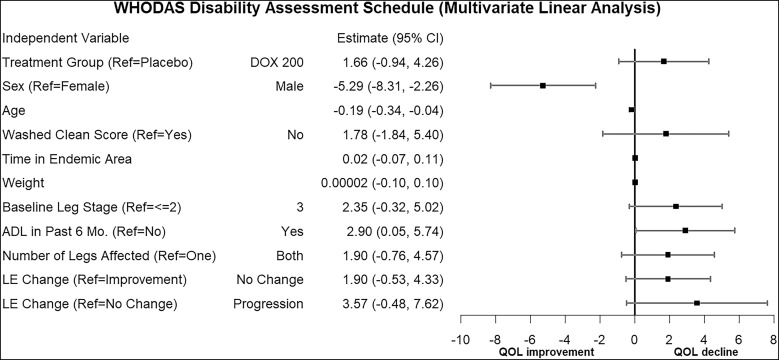
Mixed-effects linear regression analysis of factors associated with WHODAS 2 score. This forest plot graphs the point estimates and corresponding CI of the fixed effects in a mixed-effects model whose outcome was perceived disability (measured repeatedly at 6, 12, 18, and 24 months). While most independent variables had CI which crossed 0, indicating no significant effect, male sex and age had significant negative effects. This indicates that at follow-up, men reported less disability than women, and increasing age was associated with decreased perceived disability. Conversely, experiencing acute adenolymphangitis (ADL) in the last 6 months was associated with significantly increased perceived disability. DOX = doxycycline; LE = lymphedema; QOL = quality of life; Ref = reference.

**Figure 10.**
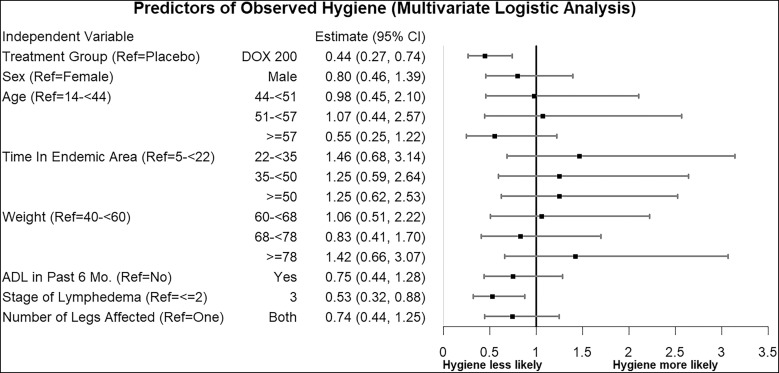
Predictors of hygiene and limb care based on physical examination. This forest plot graphs the odds ratios and corresponding CI of the fixed effects in a mixed-effects model whose outcome was observed limb hygiene across the study (measured repeatedly at 6, 12, 18, and 24 months). While the CI of most predictors crossed an odds ratio of 1, indicating no significant effect, baseline lymphedema stage and treatment group were both negatively associated with observed hygiene, with participants whose legs were stage 3 at baseline and participants who received doxycycline (DOX) being less likely to have good limb hygiene, as observed by study staff. ADL = acute adenolymphangitis; Ref = reference.

In a linear repeated-measures analysis, men and older individuals had a significantly greater reduction in WHODAS 2.0 scores, corresponding to less perceived disability (i.e., improvement in quality of life), whereas participants who had an ADL in the 6 months prior to the study experienced an increase in WHODAS 2.0 scores, corresponding to more perceived disability (Figure 9). The appearance of adherence to hygiene measures, assessed by physical examination at clinic visits, was lower in the doxycycline group and among those with stage 3 lymphedema at baseline (Figure 10).

### Treatment-emergent adverse events.

Overall, the medication was tolerated well by most participants, with only 34 adverse events being reported by 24 patients (11%), during the 6-week period of treatment with doxycycline or placebo (Table 7 shows the most common five adverse events). Most of the reported events were gastrointestinal, such as gastritis and vomiting, and were associated more with doxycycline intake. One serious adverse event occurred during treatment: a 53-year-old female patient who had previously undiagnosed Wilson’s disease suffered from hepatic encephalopathy shortly after starting doxycycline (hepatic decompensation due to broad-spectrum antibiotics is a recognized problem with Wilson’s disease). None of the other events were severe, although treatment was stopped in two more patients receiving doxycycline, one with increased liver enzymes and a facial rash and the other with increased liver enzymes, aphthous ulceration, and loose stools. A single life-threatening event (an anaphylactic reaction to amoxicillin/clavulanate given for ADL treatment) occurred during follow-up but was unrelated to the 6 weeks of study treatment.

**Table 7:** Five most frequently reported adverse events occurring during treatment

Adverse Event	Number (%) of Events		
	Doxycycline Treatment Arm	Placebo Arm	Total
Gastritis	5 (50)	5 (50)	10 (100)
Vomiting	6 (100)	0 (0)	6 (100)
Cellulitis of leg	3 (60)	2 (40)	5 (100)
Headache	3 (75)	1 (25)	4 (100)
Aphthous ulcer	1 (50)	1 (50)	2 (100)

## DISCUSSION

### Research findings.

The present study is one of a series of coordinated clinical trials aimed at confirming the preliminary finding, reported initially by Mand et al.,^12^ that 6 weeks of doxycycline treatment had a protective effect against the progression of lymphedema in patients with or without active filarial infection living in a region in Ghana where filariasis was endemic. The present study differed from the earlier one by ensuring the inclusion of concurrent adherence by all patients in the doxycycline and placebo study groups to WHO’s recommended, hygiene-focused management protocol for lymphedema. This design allowed us to ask the research question of whether a 6-week course of doxycycline brings additional benefits to the current WHO-recommended limb hygiene measures that form the basis of the EPC for lymphedema management.^25^

Unexpectedly, we observed no added benefit from doxycycline treatment compared with placebo during this 2-year study with its detailed assessments every 6 months. However, it was clear that the majority of patients in both arms (doxycycline and placebo) displayed favorable outcomes (Figure 3), namely, 1) either no progression of lymphedema throughout the study period or an improvement in the stage of lymphedema, with some participants moving from stage 3 to either stage 1 or 0 (Figure 5); 2) improvement (i.e., decrease) in WHODAS 2.0 disability scores in the first 12 months (Figure 6B); and 3) no new ADL attacks recorded during the 24-month period (Figure 6A). The deterioration in WHODAS 2.0 scores in the second year of follow-up may, in part, be due to the impact of restrictions during the pandemic.

All participants in this trial (whether on doxycycline or placebo) were repeatedly trained in hygiene methods, provided with materials required for conduct of hygiene care, and supervised by a MO who visited them at regular intervals. High adherence to hygiene procedures was observed throughout the study and was likely to have been of significant benefit to the participants. However, the lack of variability in observed hygiene meant that from a statistical perspective, it was not possible to determine the comparative impact of hygiene on outcomes commonly associated with lymphedema, especially ADL episodes. Thus, while the intensive protocol used for hygiene care might have masked any recognizable effects from the doxycycline treatment, the results do show clearly how much clinical and personal benefit can be obtained from intensive hygiene management alone. Indeed, these findings, namely, that most participants regardless of the treatment group showed a lack of lymphedema progression, an absence of ADL episodes, and even improvements in lymphedema stage over the course of the study, should be viewed as an important, clear-cut validation of the effectiveness and value of adherence to limb hygiene in lymphedema management.

In multivariate regression analysis, both the treatment group and the stage of lymphedema were significant predictors of reviewer-assessed hygiene at follow-up (*P* <0.05) (Figure 10), with stage 3 limbs appearing significantly less hygienic than those at stages ≤2, and with participants in the doxycycline treatment group being less likely to have washed and clean limbs than those in the placebo group. The minimally visible improvements occurring in this stage of disease might have discouraged patient compliance in maintaining good limb hygiene.

Older patients (≥51 years) had significantly less improvement in lymphedema than younger patients (14 to <44 years; *P* <0.05). Similarly, patients who had experienced any ADL attacks in the 6 months prior to the study had significantly less improvement than those who did not have such attacks (Figure 7) (*P* <0.05). These findings reflect the sequence of disease mechanisms that commonly occur over time, i.e., the early lymphatic damage appears as dilation associated with inflammatory mediators from both host and parasite that cause gradual impairment of lymphatic integrity and function, leading to a relative stasis of lymph that permits growth of localized pathogens that give rise to recurrent ADL attacks that can further aggravate damage to the lymphatics.^26^^,^^27^

Progression of the disease (worsening) was significantly less in those patients with stage 3 lymphedema than in those with stage ≤2 lymphedema (Figure 8). Interestingly, lymphedema progression in these patients was significantly lower despite the comparatively poor hygiene status of their limbs. Thus, it appears that disease progression (at least as measured by an admittedly imprecise stage grading scale) is more rapid in the initial stages, irrespective of limb hygiene. Since lymphatic insufficiency results in the accumulation of fluid, cells, and proteins in subcutaneous tissue spaces, with chronic inflammatory processes occurring over time and leading to changes in tissue composition and architecture (hyperkeratosis, papillomatosis, and other skin changes^28^), hygiene care is especially beneficial when initiated in the early stages of the disease.

Linear regression analysis of the WHODAS 2.0 score indicated a significantly lower impact of lymphedema on the disability scores (physical, psychological, and social) among male patients with lymphedema (Figure 9), and such sex variability should be appreciated when implementing programs for disability management and rehabilitation.

### Trial conduct.

All participants of the LEDoxy Sri Lanka clinical trial were very cooperative throughout the entire study period. They were pleased to be part of the trial and were eager to know whether they had received the drug or the placebo. Those patients who had obvious improvement in lymphedema were looking forward to receiving further doses of medication in hopes of achieving a further reduction or even a complete cure. All study participants enjoyed the individual attention they received throughout the 2-year period of this trial and were somewhat reluctant to go back to visiting their regular health clinics, as they felt they would not receive similar support.

This LEDoxy Sri Lanka trial managed to continue without disruption amid all hardships of the COVID-19 pandemic. Patients were contacted, and private transport was arranged for them to attend follow-up clinics as scheduled, since public transport was largely dysfunctional. However, some participants who were located far away had to cross district borders and were not permitted to visit the clinic during the lockdown period. Therefore, arrangements were made for two remote clinics to be established (in Dickwella and Matara) and to receive the participants who were transported in small groups using a study vehicle. The study team, including the principal investigator, participated in all clinic follow-up visits, with appropriate safety precautions ensured during these visits to avoid infection and transmission of the virus.

## CONCLUSION

The apparent lack of benefit from the addition of a 6-week treatment with doxycycline to the hygiene-based EPC is most likely due to the enhanced hygiene measures implemented throughout the study period. Additionally, in contrast to the original Ghana studies that took place in areas with active LF transmission, in the Sri Lanka study sites, there was little or no active, ongoing LF transmission, as evidenced both by the serologic evaluations of the study patients and by WHO’s recognition of the success of Sri Lanka’s LF elimination program.^3^ The findings of this study reinforce the precept that rigorous adherence to limb hygiene, especially in the early stages of LF, is an essential element in preventing lymphedema progression.

## Data Availability

Data availability statement: The data that support the findings of this study are openly available in ClinEpiDB at https://clinepidb.org/ce/app/workspace/analyses/DS_f71667fc7f.
